# Mapping Fluorescence Enhancement of Plasmonic Nanorod Coupled Dye Molecules

**DOI:** 10.3390/nano10061048

**Published:** 2020-05-29

**Authors:** Emese Tóth, Ditta Ungor, Tibor Novák, Györgyi Ferenc, Balázs Bánhelyi, Edit Csapó, Miklós Erdélyi, Mária Csete

**Affiliations:** 1 Department of Optics and Quantum Electronics, University of Szeged, Dóm Square 9, H-6720 Szeged, Hungary; tothemese@titan.physx.u-szeged.hu (E.T.); novaktibor@titan.physx.u-szeged.hu (T.N.); erdelyim@physx.u-szeged.hu (M.E.); 2 Department of Physical Chemistry and Materials Science, Interdisciplinary Excellence Centre, University of Szeged, Rerrich B. Square 1, H-6720 Szeged, Hungary; ungord@chem.u-szeged.hu (D.U.); juhaszne.csapo.edit@med.u-szeged.hu (E.C.); 3 Institute of Plant Biology, Biological Research Centre, Temesvári krt. 62, H-6726 Szeged, Hungary; ferenc.gyorgyi@brc.hu; 4 Department of Computational Optimization, University of Szeged, Árpád Square 2, H-6720 Szeged, Hungary; banhelyi@inf.u-szeged.hu; 5 MTA-SZTE Biomimetic Systems Research Group, Department of Medical Chemistry, Faculty of Medicine, University of Szeged, Dóm Square 8, H-6720 Szeged, Hungary

**Keywords:** gold nanorod, plasmon resonance, Cy5 dye molecule, enhanced fluorescence, optimization, DNA, STORM

## Abstract

Plasmonically enhanced fluorescence is a widely studied and applied phenomenon, however, only a comparative theoretical and experimental analysis of coupled fluorophores and plasmonic nanoresonators makes it possible to uncover how this phenomenon can be controlled. A numerical optimization method was applied to design configurations that are capable of resulting in an enhancement of excitation and emission, moreover, of both phenomena simultaneously in coupled Cy5 dye molecule and gold nanorod systems. Parametric sensitivity studies revealed how the fluorescence enhancement depends on the molecule’s location, distance and orientation. Coupled systems designed for simultaneous improvement exhibited the highest (intermediate directional) total fluorescence enhancement, which is accompanied by intermediate sensitivity to the molecule’s parameters, except the location and orientation sensitivity at the excitation wavelength. Gold nanorods with a geometry corresponding to the predicted optimal configurations were synthesized, and DNA strands were used to control the Cy5 dye molecule distance from the nanorod surface via hybridization of the Cy5-labelled oligonucleotide. State-of-the-art dSTORM microscopy was used to accomplish a proof-of-concept experimental demonstration of the theoretically predicted (directional) total fluorescence enhancement. The measured fluorescence enhancement was in good agreement with theoretical predictions, thus providing a complete kit to design and prepare coupled nanosystems exhibiting plasmonically enhanced fluorescence.

## 1. Introduction

The plasmon enhanced fluorescence phenomenon is a synergetic action of the excitation rate enhancement and the modification of the overall quantum yield, and is affected by the achievable out-coupling efficiency as well.

In the primary literature of fluorescence improvement, the excitation enhancement was computed based on the **E**-field enhancement around a dipolar emitter, whereas the emission enhancement was treated by a surface induced radiative rate correction [1,2]. Accordingly, the fluorescence intensity enhancement of a plasmonic antenna coupled emitter was usually computed as δ*F* = δ*γ_exc_* × δ*η_em_* × δ*ε_col_*_l_, namely the excitation enhancement was rescaled by the quantum efficiency modification at the emission, and the collection efficiency improvement via plasmonic antenna-like resonators was also taken into account [3,4]. It was proven that the involved antenna modes determine the relative strength of radiative and non-radiative rates, and quenching/fluorescence enhancement occurs typically on the blue/red side of the spectral peaks corresponding to resonances on plasmonic nano-objects [5].

Primary studies predicted that the excitation and emission improvement can be compromised, when the single plasmon resonance peak is in between the corresponding wavelengths [6,7]. On nanorods, the co-existent transversal and longitudinal resonances are beneficial, however, as a cost of strong polarization dependency [8]. The difference between the Purcell factor (total decay rate enhancement) and radiative rate enhancement was attributed to losses in the coupled fluorophore-nanophotonic systems [9]. Good scattering efficiency was demonstrated in the case of core-shell particles and tapered nanoantennas (spheres and tori) aligned above films, whereas directional out-coupling and large degrees of freedom are the advantages of dimer antenna arrays [10,11,12,13]. The existence of an optimal distance to promote dye molecule and nanoresonator coupling was also revealed [10,14]. In our previous studies, a novel theoretical approach was developed to extract the total and radiative decay rate enhancements both at the excitation and emission wavelengths, and to determine the optimal configurations for arbitrary coupled fluorophore and plasmonic nanoresonator systems [15,16,17].

In experimental studies, a single molecule and single plasmonic nano-object interaction was preferred, since in the case of multitudes, the plasmonic antennas density has to be larger than a threshold, but their size distribution makes the plasmonic responses spectrally broad and the resulted peaks are inherently blurred, caused by the random orientation of fluorophores [18]. A DNA origami was used to position a single Cy5 dye molecule and to map the local density of states (LDOS) in plasmonic nanoparticle on mirror type (NPonM) nanoresonators with a 1.5 nm resolution. It was shown that the coherent coupling of an emitter and a plasmonic nanoresonator results in a modulation of the scattering spectrum [19].

Extended studies on coupled emitter and plasmonic nano-object systems created a great demand for high spatial resolution microscopy techniques. Diffraction–limited resolution can be surpassed, for example, by stimulated emission depletion (STED) [20], stochastic optical reconstruction microscopy (STORM) [21] and photo-activation localization microscopy (PALM) [22] that are capable of resulting in single-molecule localization (SML) as well.

Three phenomena can improve SML precision in close proximity of metal surfaces: (i) the metal quenches the molecules adsorbed on it that provides a contrast to dielectric objects, (ii) excitation enhancement is also achieved at the (localized) surface plasmon resonances ((L)SPR), and (iii) the stability of the molecules is improved, since due to the increased decay, the lifetime in excited states is decreased, and the probability that the emitter undergoes photo-induced chemical processes is decreased as well [9,23]. Accordingly, resolution enhancement was achieved via surface plasmon resonance illumination [24]. Novel microscopy methods appeared as well, e.g., Brownian motion single molecule super-resolution imaging [25], moreover, several former microscopy techniques were developed by using plasmonic nanoparticles, e.g., in STED [26].

It was shown that the apparent position of a dipolar emitter in close proximity of a plasmonic antenna is governed by a distance dependent partial coupling due to the decay of the molecule through two competing radiative pathways: direct and mediated by the antenna [27]. Moreover, it was described that the apparent position of emitters coupled to metal nanorods can be affected by the objective induced point-spread function (PSF) aberrations [28].

After excluding the effect of dye concentration, quality, molecule distance and image dipole formation, the apparent smaller nanorod size was attributed to heterogeneous binding of double-strand DNA used as a linker [29]. This indicates that there exists a great demand for predesigned DNA linkers’ development.

In the case of STORM, the advantage of controlled emission detuning from SPR via a large Stokes shift was proven, since it promoted large localization precision and high-resolution electromagnetic-field mapping, whereas fluorophores coupled at their emission made it possible to explore the LDOS distribution with high-resolution [30].

The synergy between super-resolution imaging and plasmonics can result in significant resolution improvement, and accordingly, plasmonic NPs can be used as image contrast agents, whereas plasmonically tailored excitation fields can promote sub-diffraction-limited spatial resolution, e.g., in STED [31]. The optimal spectral and spatial conditions to improve the super-resolution imaging of plasmonic particles correspond to a partial decoupling, which makes it possible to map the realistic nano-object shape [32,33]. This has been demonstrated via motion blur point accumulation for imaging in nanoscale tomography (PAINT) and via DNA-PAINT as well. Single molecule microscopy and spectroscopy proved that photon count rate enhancement in the order of 10–10^2^ is achievable at small distances defined by DNA strands, when the longitudinal resonance of the nanorod is tuned to the excitation and emission wavelength [34]. Confocal microscopy proved that the selective attachment of dye molecule assemblies at DNA strand-controlled distances into plasmonic hot spots of nanorods results in 10-fold fluorescence enhancement, when the LSPR overlaps with the emission band [35]. However, systematic computations and microscopy on a single fluorescent molecule coupled to a single NP exhibiting resonance at a spectral location exactly in between the excitation and emission wavelengths has not been reported previously.

In our present study, coupled Cy5 dye molecule and gold nanorod (Au NR) systems have been designed via numerical computations to improve excitation and emission simultaneously. Analogue Cy5–Au NR systems were prepared by controlling their distance via double-strand DNA and then inspected via dSTORM. The fluorescence responses detected by dSTORM have been compared to the (directional) fluorescence enhancement that is achievable via coupled Cy5 dye molecule and gold nanorod configurations optimized by special numerical computation methods.

## 2. Methods

### 2.1. FEM Optimization and Analyses of Optimal Configurations

In our previous studies, we have shown that the radiative rate enhancement (δ*R*) of a dipolar emitter can be determined by multiplying the Purcell factor (which is the total decay rate enhancement in a plasmonic nanoresonator with respect to a homogeneous environment) and the quantum efficiency (*QE*, which is the ratio of the power radiated into the far-field to the total emitted power) at the wavelength either of excitation or emission [15,16,17]:

*Purcell factor* = *P*_total_/*P*_total_0_ = (*P*_radiative_ + *P*_non-radiative_)/(*P*_radiative_0_ + *P*_non-radiative_0_) (1)

*QE* = *P*_radiative_/(*P*_radiative_ + *P*_non-radiative_) (2)

*δR* = *Purcell factor* × *QE* (3)

In coupled systems consisting of a fluorophore and a plasmonic nanoparticle, a significant part of the emitted power is transferred to the NP, however, only a fraction of it escapes to the far-field, whereas the remaining part is lost in the form of resistive heating. This explains why optimization is necessary to determine configurations that are the most appropriate for fluorescence enhancement.

The coupled systems were modelled by using the RF module of the *COMSOL Multiphysics* software package (version 5.5; COMSOL AB, Stockholm, Sweden, 2019). Single gold nanorod coated with a DNA strand was deposited on an NBK7 substrate in an aqueous environment. The single Cy5 dye molecule was modelled as a dipolar emitter of a variable oscillation frequency that was the source of the reference and coupled system as well.

**Figure 1:**
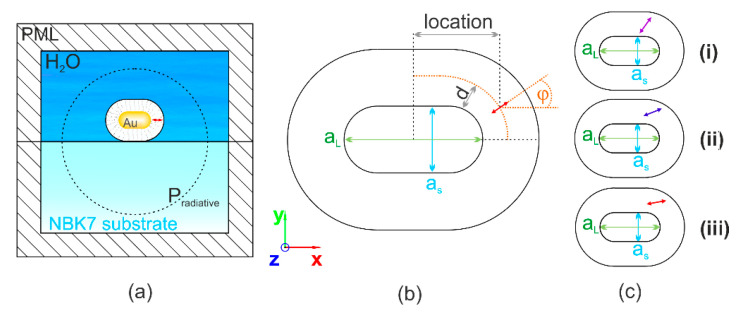
(**a**) Extraction methodology of quantities used to evaluate the coupled Cy5 dye molecule–gold nanorod systems. (**b**) Schematic drawing of the coupled system parameter’s variation: dipole location (*x*) along the long axis of and distance (*d*) from the nanorod, as well as orientation (*φ*) with respect to the long axis of the nanorod. (**c**) Schematics of the configurations received via i-ii-iii type optimizations.

The model consisting of a single Cy5 dye molecule and a single Au NR coupled system on a substrate was terminated by an absorbing boundary condition with a perfectly matched layer (PML) layer (Figure 1a). The mesh size was smaller than the skin-depth/five on the gold nanorod, and *λ*/(8 × *n*) in the media of wave propagation.

The geometry of the inspected gold nanorods has been characterized by their short (*a*_s_) and long (*a_l_*) axes, and based on these parameters, the aspect ratio (*AR*) has been also computed (Figure 1b,c). The dipolar emitter modelling of the Cy5 dye molecule was characterized by the location (*x*), specifying the distance of the dipole from the center along the long axis of the nanorod (that is parallel to the *x* axis); distance (*d*), specifying the distance of the dipole from the nanorod surface inside the DNA strand having a fixed thickness of 17.5 nm; orientation (*φ*), specifying the tilting angle of the dipole measured with respect to the long axis of the nanorod (Table 1, Figure 1b,c). Plasmon coupled emission was inspected for dipolar emitters lying in the *xy* plane, which ensures a better comparison with the experimental results, taking into account the dSTORM configuration, where light propagating along the z axis and incident perpendicularly to the substrate is used for excitation, whereas emission is collected from a complete half space in the TIRF mode from randomly distributed and oriented Cy5 dye molecules.

The extraction of the Purcell factor, *QE* and δ*R* has been performed by applying the method described in our former paper [15]. The modification of the directivity (δ*D = D/D_0_,* where *D* and *D*_0_ are the directivity of the coupled Cy5—Au NR system and uncoupled Cy5 dye molecule, respectively) has been evaluated to determine the coupled fraction of enhanced emission (δ*D* × δ*R*). At the excitation, the directivity was computed by supposing an exciting beam that is perpendicularly incident onto the substrate and is polarized along the *x* axis, i.e., along the long axis of the gold nanorod. At the emission, outgoing beams into all directions were taken into account, independently of polarization, to ensure comparability with the dSTORM measurements. Finally, the total fluorescence enhancement, which is qualified by the *P_x_ factor* = δ*R*_exc_ × δ*R*_em_, as well as the fraction of the total fluorescence enhancement that can be captured via dSTORM microscopy (*D_x_ factor* = *P_x_ factor* × δ*D*_exc_ × δ*D*_em_) have been evaluated for all of the optimized systems (Table 2).

#### 2.1.1. Optimization of Cy5 Dye Molecule and Gold Nanorod Coupled Systems

The excitation (635 nm) and emission (665 nm) wavelengths of the inspected Cy5 dye molecule are close to each other, so it is expected that both phenomena can be enhanced by a single broad dipolar plasmonic resonance of a predesigned gold nanorod. Accordingly, during optimization, the Purcell factor, quantum efficiency and radiative rate enhancement of the coupled systems have been monitored at both wavelengths. The intrinsic quantum efficiency (~25%) of the Cy5 dye molecule is low, therefore, *QE* improvement is desired as well.

Three sets of optimizations have been performed by applying the following objective functions: (i) radiative rate enhancement at the excitation (δ*R*_exc_), (ii) radiative rate enhancement at the middle of the spectral interval between excitation and emission (δ*R*_[exc,em]/2_), and (iii) radiative rate enhancement at the emission (δ*R*_em_). The conditional optimization accepted those configurations, which met the *QE*_em_crit_ = 25 ± 0.25% criterion regarding the quantum efficiency at the emission, and exhibited a maximum that appeared at the required wavelength on the δ*R* spectrum.

The geometrical parameters of the gold nanorods were varied in the interval of the short and long axes that were determined in the preliminary TEM measurements (Figure 1, Table 2, please see Section 2.2.3 and Section 3.2). In order to ensure overlapping with the experimental parameters, the *a*_s_ short axis of the Au NRs was varied in the interval of [12.1 nm, 22.1 nm], whereas the AR was allowed to take on values in the interval of [1.85, 4.25]. Additionally, the parameters of dipolar emitters corresponding to Cy5 dye molecules have been varied in the intervals determined by considering the gold nanorod geometry under optimization and the fixed 17.5 nm DNA strand thickness. Namely, the dipole location (*x*) could take on values in the interval of [0, *a*_l_/2+*d*]; the dipole distance (*d*) was allowed to vary in the [8.9 nm, 17.5 nm] interval; whereas the dipole orientation (*φ*) could take on random values in the [0, π/2] interval (Figure 1b).

The GLOBAL method was used to optimize the coupled Cy5 dye molecule and gold nanorod configurations. GLOBAL is a stochastic optimization algorithm developed for hard, unconstrained problems with a few local optima and is robust also for a large number of variables [36]. The penalty approach was used to define the conditions for allowed geometry and *QE* parameters and to find maxima on the spectra. The achieved radiative rate enhancement spectra were sampled in 5 nm discrete steps in order to find a maximum.

#### 2.1.2. Analyses of Optimal Cy5 Dye Molecule and Gold Nanorod Coupled Systems

**Figure 2:**
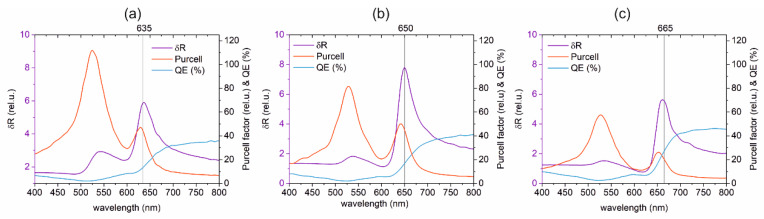
Purcell factor, quantum efficiency (*QE*) and radiative rate enhancement (δ*R*) spectra of the coupled systems optimized to maximize (**a**) excitation, (**b**) excitation and emission simultaneously and (**c**) emission of Cy5 dye molecule.

**Figure 3:**
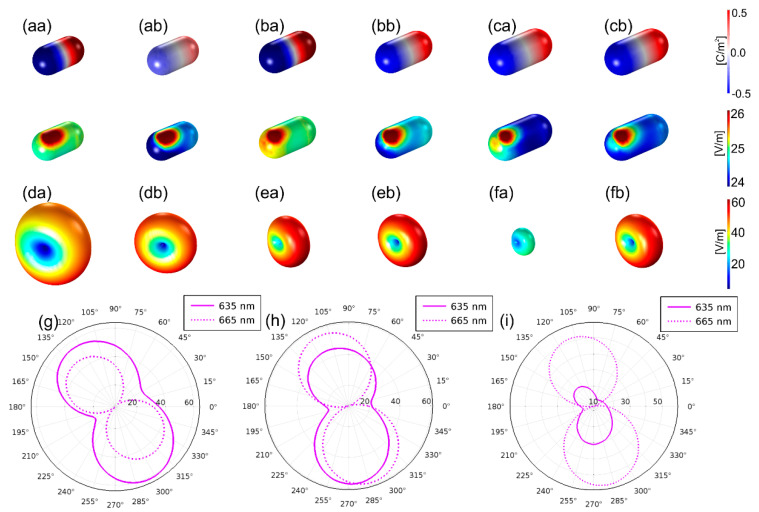
Near-field and far-field response of the optimized systems: (**a**–**c**) normalized **E**-field and charge distribution, (**d**–**f**) far-field radiation pattern at (**a**–**f**/**a**) excitation and (**a**–**f**/**b**) emission, (**g**–**i**) polar diagram at the excitation and emission wavelengths (continuous and dashed line) of the system optimized to enhance (**a**,**d**,**g**) excitation, (**b**,**e**,**h**) excitation and emission simultaneously and (**c**,**f**,**i**) emission of Cy5 dye molecule.

The wavelength dependence of the Purcell factor, quantum efficiency and radiative rate enhancement has been determined for the optimal configurations received in the (i)–(iii) optimization cases (Figure 1c or Figure 2a–c). In these computations, the wavelength of the dipolar emitter modeling the Cy5 dye molecule has been tuned. The charge and near-field distribution (Figure 3a–c) as well as the far-field radiation pattern (Figure 3d–f or g–i) have been analyzed in the optimal configurations of the (i)-, (ii)- and (iii)-type coupled systems as well.

#### 2.1.3. Sensitivity Study on Cy5 Dye Molecule and Gold Nanorod Coupled Systems

**Figure 4:**
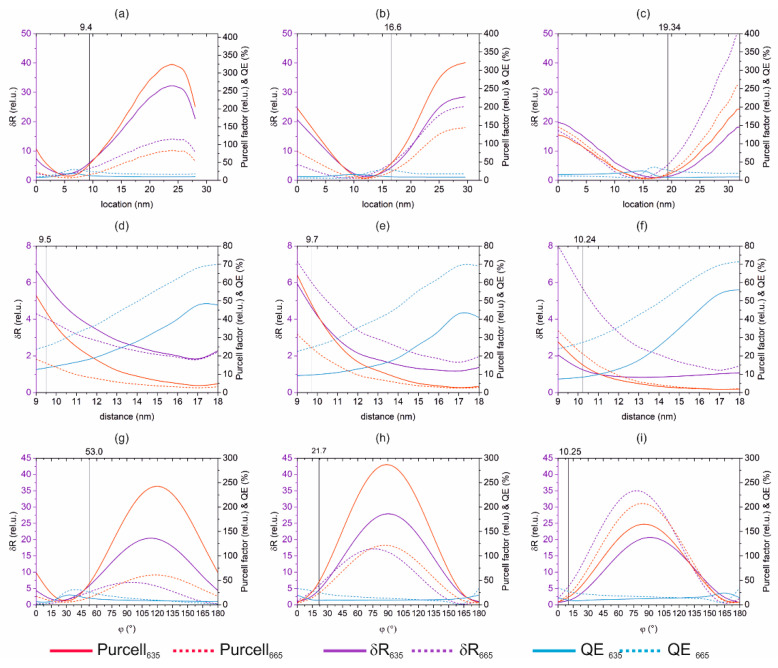
Dependence of the achievable Purcell factor, quantum efficiency (*QE*) and radiative rate enhancement (δ*R* = *Purcell × QE*) on the (**a**–**c**) dipole location, (**d**–**f**) distance from the nanorod inside 17.5 nm thick DNS strand, (**g**–**i**) orientation of the Cy5 dye molecule, whereas the other two parameters are constant in the (*x*_opt_ location, *d*_opt_ distance, *φ*_opt_ orientation) parameter set in the case of coupled systems optimized to enhance (**a**,**d**,**g**) excitation, (**b**,**e**,**h**) excitation and emission simultaneously and (**c**,**f**,**i**) emission phenomenon of Cy5 dye molecule.

It was studied how the achievable Purcell factor, *QE* and radiative rate enhancement varies, when the location of the Cy5 dye molecule is tuned from the center through the apex of the gold nanorod at the optimal distance and orientation (Figure 4a–c), and when the distance of the Cy5 dye molecule from the gold nanorod surface is increased at the optimal location and orientation (Figure 4d–f). The dipole orientation dependence of the Purcell factor, *QE* and radiative rate enhancement has been inspected as well at the optimal location and distance of the Cy5 dye molecule inside the (i)-, (ii)- and (iii)-type coupled systems (Figure 4g–i).

In our present study, dipolar emitters aligned in the *xy* plane have been analyzed, however, similar behavior is expected in the *xz* and *yz* plane, and an additional comparative study on them will be presented in an upcoming theoretical work.

### 2.2. Preparation Protocol and Characterization of Au NRs

#### 2.2.1. Materials Used to Prepare Au NRs

All chemicals were of analytical grade and were used without further purification. The gold (III) chloride acid trihydrate (HAuCl_4_∙3H_2_O; ≥99.9%) and the hexadecyltrimethylammonium bromide (CTAB, C_16_H_33_N(CH_3_)_3_Br, ≥98%) were from Sigma (St. Louis, MO, USA). The hydrogen chloride solution (HCl, 37%), the sodium borohydride (NaBH_4_; 99%) and the silver nitrate (AgNO_3_; ≥98%) were from Molar (Budapest, Hungary).

#### 2.2.2. Preparation Protocol of Au NRs

The Au NRs were synthesized by a modified soft template method [37]. Firstly, the seed solution was prepared by adding 250 µL 0.01 M HAuCl_4_ to 10.0 mL 0.1 M CTAB solution. Upon the appearance of the dark orange color, freshly prepared ice-cold NaBH_4_ (0.6 mL 0.01 M) solution was quickly added into the mixture and stirred for 5 min. The formation of the brown color refers to the formation of gold seeds in the solution. The gold colloids were kept at room temperature for at least 2 h before using them. For the seed-mediated growth, 2.0 mL 0.01 M HAuCl_4_ and the 150 µL of 0.01 M AgNO_3_ were compounded with 40 mL 0.1 M CTAB solution. After the mixing, 0.32 mL 0.1 M freshly prepared ascorbic acid, 0.8 mL 1M HCl and 96 µL seed solution were added into the solution. The reaction mixture was blended by gentle shaking. Finally, the growing solution was left undisturbed for 24 h at 40 °C.

#### 2.2.3. Characterization of the Synthesized Au NRs

**Figure 5:**
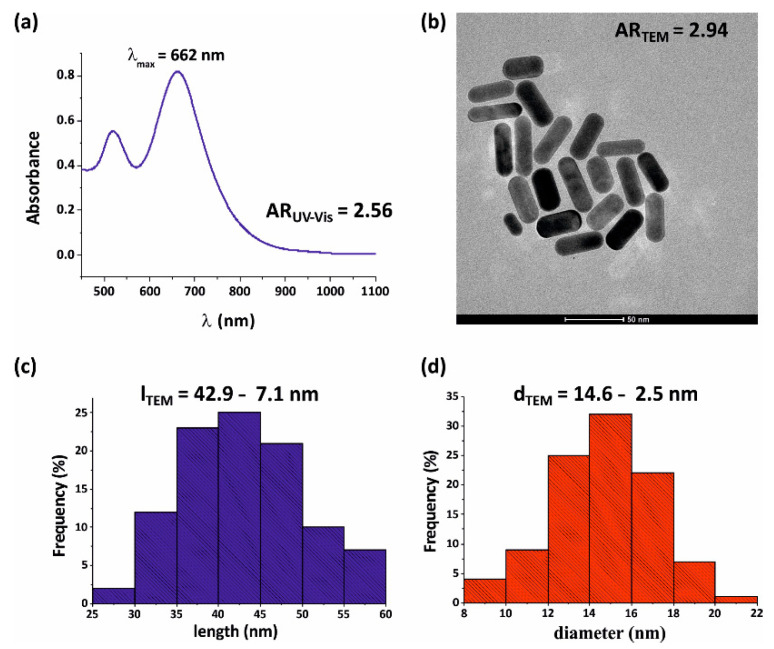
The UV–Vis spectrum (**a**) and the TEM image (**b**) of the chemically synthesized Au nanorods (NRs). The long (**c**) and short (**d**) axes distributions of the prepared Au NRs calculated based on TEM images.

For the optical characterization, the UV–Vis–NIR spectra were recorded on a JASCO, V-770 Spectrophotometer (Budapest, Hungary) using 1 cm quartz cuvette from 190 to 1000 nm. High-resolution transmission electron microscopy (HRTEM) images were captured on a FEI Technai G2 instrument (Hillsboro, OR, USA) at 200 kV accelerating voltage. The samples were dried on carbon film Cu grids (Electron Microscopy Sciences, 200 mesh; Hatfield, PA, USA). The images were analyzed by the *ImageJ* software (Version 1.52t, open platform by LOCI, University of Wisconsin, USA; 2020). The aspect ratio of the synthesized Au NRs was determined based on the TEM images and on the UV–Vis spectra [38] (Figure 1 and Figure 5).

### 2.3. Synthesis and Purification of Thiol- and Cy5-Modified DNA Oligonucleotides, Functionalization of Au NRs with Thiol-Modified DNA Oligonucleotide and Hybridization of the Cy5-Labelled Oligonucleotide

#### 2.3.1. Materials Used to Create DNA Strands

An in-house synthesized 4,4′,4″-trimethoxytrityl (TMTr)-protected 5′-Thiol-Modifier C6 phosphoramidite was used [39]. Phosphoramidites, synthesis reagents and solvents used for DNA oligonucleotide synthesis were purchased from Sigma-Aldrich Kft. (Budapest, Hungary), Molar Chemicals Kft. (Budapest, Hungary) and LGC Genomics Ltd. (Teddington, UK).

#### 2.3.2. Synthesis and Purification of Thiol and Cy5-Modified DNA Oligonucleotides

DNA oligonucleotides were synthesized using a DNA/RNA/LNA H-16 synthesizer (K&A Laborgeraete, Schaafheim, Germany) by standard β-cyanoethyl phosphoramidite chemistry at a nominal scale of 0.2 μmol. Sequences of the complement oligonucleotides are included in Table 1.

Oligonucleotides were purified by HPLC on an RP-C18 column under ion-pairing conditions (using mobile phases containing 0.1 mM triethylammonium acetate (TEAA), pH 6.5 buffer). The 4,4′-dimethoxytrityl and 4,4′,4″-trimethoxytrityl protective groups from the 5′-end of the oligonucleotide were removed using 2% TFA on a Poly-Pak column (Sterling, VA, USA). After lyophilisation, 100 µM stock solution was prepared from the oligonucleotides.

#### 2.3.3. Functionalization of Au NRs with Thiol-Modified DNA Oligonucleotide

Functionalization of Au NRs with thiol-modified-DNA was done based on mPEG-SH (linear monofunctional PEG with a reactive free thiol, SH, MW ~5 kDa)/Tween 20-assisted method in order to avoid aggregation of the positively charged Au NRs and negatively charged DNA [40].

Using this fast method, in 1 h, Tween 20 and mPEG-SH displaced CTAB on the surface of Au NRs and 5’-thiol-modified-DNA can be attached to it in a 100 mM solution of sodium-citrate.

Accordingly, Au NRs were functionalized based on Jiuxing Li et al.’s method with the following modifications: (1) mPEG-SH was added two times [39]; (2) after 1 h, aging DNA-Au NRs were washed two times with 20 mM HEPES (4-(2-hydroxyethyl)-1-piperazineethanesulfonic acid) buffer (pH 7.1) and after final centrifugation, were resuspended in 20 mM HEPES (pH 7.1) with 50 mM NaCl (100 µL).

#### 2.3.4. Hybridization of the Cy5-Labelled Oligonucleotide to the DNA-Au NR

In the following step, a 10 µL solution of Cy5-labelled complement DNA (in 100 µM concentration) was added to the 100 µL DNA-Au NR solution in 20 mM HEPES with 50 mM NaCl, and incubated at 65 °C for 5 min, then gradually brought to room temperature (−2 °C/min). Unbound oligonucleotide was removed by centrifugation and the Au NR functionalized with Cy5-labelled DNA duplexes was resuspended in 20 mM HEPES (pH 7.1) and it was stored at 4 °C until further use [41]. Several Cy5 dye molecule distances in the interval of [0 nm, 17.5 nm] were defined inside DNA strands having a thickness of 17.5 nm. In the microscopy measurements detailed in the present paper, the Cy5 dye molecule distance of 8.9 nm has been applied.

### 2.4. dSTORM Optical System, Sample Preparation, Measurement and Analysis

#### 2.4.1. dSTORM Optical System

Super-resolution dSTORM measurements were performed on a custom-made inverted microscope based on a Nikon Eclipse Ti-E frame (Nikon Instruments Europe BV, Amsterdam, The Netherlands). After being conditioned (through spatial filtering via fiber coupling and beam expansion), the applied laser beams were focused onto the back focal plane of the microscope objective (Nikon CFI Apo 100×, NA = 1.49), which produced a collimated beam on the sample. All dSTORM images were captured with a linearly polarized beam and EPI illumination at an excitation wavelength of 647 nm (MPB Communications Inc., Pointe-Claire, QC, Canada: 647 nm, *P*_max_ = 300 mW). The laser intensity was controlled via an acousto-optic tunable filter (AOTF). Images were captured by an Andor iXon3 897 EMCCD camera (Andor: Belfast, UK; 512 × 512 pixels with 16 μm pixel size). Frame stacks for dSTORM super-resolution imaging were typically captured at a reduced image size (crop mode). Excitation and emission wavelengths were spectrally separated with a fluorescence filter set (Semrock, Rochester, NY, USA; LF405/488/561/635-A-000) and an additional emission filter (Semrock, BLP01-647R-25) in the detector arm. During the measurements, the perfect focus system of the microscope was used to keep the sample in focus with a precision of <30 nm.

#### 2.4.2. dSTORM Sample Preparation

**Figure 6:**
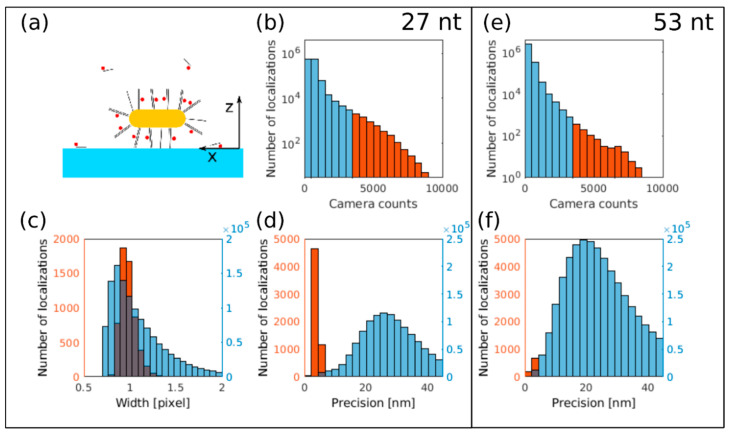
(**a**) Schematic figure of a functionalized Au nanorod on the coverslip. Localizations with a brightness value higher than 3500 camera counts were selected for further evaluations using the (**b**) 27 nt and (**e**) 53 nt long spacers. The intense localizations correspond to the (**c**) in focus localizations with full width at half maximum (FWHM) ≈ 1 pixel and to the most precisely (<6 nm) localized ones using the (**d**) 27 nt and (**f**) 53 nt long spacers. Localizations marked with orange in Figures (**b**–**f**) belong to the same group.

Cover slips were plasma treated and coated with a PAH (poly(allylamine hydrochloride)) cationic polyelectrolyte monolayer. The solution of the functionalized gold nanorods was dropped on the cleaned glass cover slips. After five minutes of sedimentation time, the cover slip was rinsed with pure water (W3513 BioPerformance Certified, Sigma-Aldrich Kft, Budapest, Hungary) to reduce the number of unbound oligomers. Right before the measurement, the water was replaced with glucose oxidase (GLOX)switching buffer [42] and the sample was mounted onto a microscope slide (Figure 6a).

#### 2.4.3. dSTORM Measurement

Before the dSTORM image acquisition, the positions of the Au nanorods were identified with cross-polarized white light illumination. Then, the selected areas were checked via the fluorescence mode at a low excitation power. This procedure ensured that the selected areas contained labelled nanorods as both the scattered light signal of the plasmonic nanoparticles and the fluorescence signal of the dye molecules were detected. For the dSTORM measurement, the excitation intensity was raised to 3 kW/cm^2^, and 20,000 frames were captured with a cropped area size and an exposure time of 105 × 105 pixels and 30 ms, respectively.

#### 2.4.4. dSTORM Analysis

**Figure 7:**
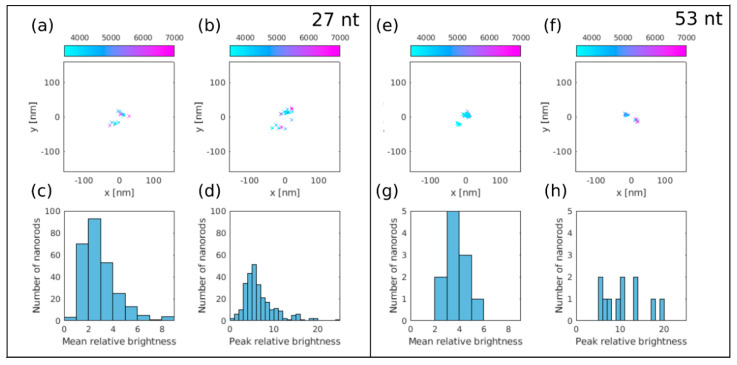
dSTORM images of two gold nanorods labelled with Cy5 dye molecules using (**a**,**b**) 27 nt and (**e**,**f**) 53 nt long spacers. The color code shows the peak counts of localizations with enhanced brightness. (**c**–**g**) Mean and (**d**–**h**) maximum fluorescence enhancement values for both spacers shows the significant difference between the identified Au NRs (260 versus 11).

The captured and stored image stacks were evaluated and analyzed with the rainSTORM localization software [43] that fitted a Gaussian point spread function to the images of the individual fluorescent Cy5 dye molecules. Localizations belonging to the same blinking event were concatenated with the trajectory fitting algorithm of rainSTORM. The acquired trajectories were used to determine the peak intensities of the PSFs of the blinking events by calculating the average of the intermediate localizations of the given trajectory. Therefore, all trajectories shorter than three frames were automatically discarded. The background of this strong restriction is based on the assumption that the ON-state lifetime of the enhanced fluorophores increases, since the S1 → S0 fluorescence path is more preferable than the S1 → T1 relaxation [44]. In the dSTORM method, the molecules can be turned off via their T1 state [42], therefore, their ON-state lifetime will increase (Figure 7).

## 3. Results

### 3.1. Optimized Coupled Cy5–Au NR Configurations

#### 3.1.1. Optical Response of the Optimized Configurations

The geometrical parameters are similar in the (i)-, (ii)- and (iii)-type optimized coupled systems (Table 2). The optimal *a*_l_ long axis gradually increases with increasing the wavelength, where the radiative rate enhancement was maximized. However, the optimal *a*_s_ short axis increases slightly as well, and the optimal *AR* aspect ratio increases gradually, suggesting these tendencies altogether ensure the forward shifting of the resonance wavelength to the desired wavelength.

The optimal location of the plasmonically enhanced Cy5 dye molecule corresponds to a gradually increasing *x* coordinate, the distance from the nanorod increases as well, but at the same time, the optimal tilting angle with respect to the long axis gradually decreases. These tendencies are in accordance with intuitive expectations based on the previously described plasmon enhanced emission phenomena in similar coupled systems [35]. All geometrical parameters take on an intermediate value in the system optimized to enhance the excitation and emission phenomena simultaneously.

The optimizations performed with the δ*R* radiative rate enhancement objective functions at different central wavelengths resulted in a coupled Cy5 dye molecule and gold nanorod systems, which exhibit a local maximum on the Purcell factor slightly below the desired wavelength, whereas the *QE* quantum efficiency shows an increasing tendency, and as a result, a global maximum appears on the δ*R* radiative rate enhancement spectrum at excitation (635 nm), exactly in between the excitation and emission (650 nm) and at the emission (660 nm), respectively (Figure 2a–c).

The total fluorescence enhancement achievable in the (i)- (ii)- (iii)-type optimized coupled systems is in the order of 10, which is in accordance with earlier results in the literature [10,14]. The *P*_x_ factor exhibits a maximum in the case of the (ii)-type optimized coupled system, proving that it is reasonable to propose it as a representative optimal configuration capable of enhancing both phenomena simultaneously. Considering the total fluorescence enhancement, the optimization performed at the mean value of the excitation and emission wavelengths is capable of resulting in a coupled system, where the two phenomena are simultaneously maximized rather than only compromised (Figure 2b, Table 2). This result is in contradiction with some earlier literature [6,7], but is in agreement with the spectral dependence of enhancement predicted for the averaged orientations of dye molecules in plasmonic hot spots of nanorods, which was computed by tuning the excitation wavelength [35].

Taking into account the directivity modification in the case of Cy5 dye molecules lying in the xy plane, the fraction of the total fluorescence enhancement that could be collected from the substrate side is approximately 98% and 51% in the (i)- and (ii)-type coupled systems, whereas only 32% of the total fluorescence enhancement is detectable in the (iii)-type system. The gradually decreasing *D*_x_ factor quantifying the directional total fluorescence enhancement indicates that the out-coupling efficiency decreases, when the target wavelength increases.

All optimized coupled systems result in a local field enhancement via dipolar modes on the gold nanorods both at the excitation and emission wavelength (Figure 3a–c). The coupled system optimized to enhance the excitation and emission phenomenon exhibits a stronger local field enhancement and larger far-field lobes at the excitation and emission wavelengths. In the system optimized to enhance these phenomena simultaneously, commensurate lobes prove intermediate enhancement simultaneously at both wavelengths in accordance with the expectations (Figure 3a,d,g/b,e,h/c,f,i).

#### 3.1.2. Optical Response Dependence on Dipole Location, Distance and Orientation

A sensitivity study uncovered how the optical response is influenced in realistic systems, where the distance of the Cy5 dye molecule can be tailored via the presently developed method of Cy5-labelled oligonucleotide hybridization to the DNA–Au NR. One has to note that the dipole location would be hard to control experimentally, and the coupled Cy5 dye molecules orientation is random. However, by comparing the computed and experimentally achieved enhancement values, it is possible to consider whether the realistic coupled system corresponds with the predesigned one.

The dipole location, distance and orientation were varied sequentially with respect to the optimal (*x*_opt_ location, *d*_opt_ distance, *φ*_opt_ orientation) parameter set. By varying the location of the Cy5 dye molecule, namely by increasing the distance from the center of the gold nanorod, all of the Purcell factor, *QE* and radiative rate enhancement non-monotonously vary (Figure 4a–c). At the optimal distance, the *QE* meets the criterion set at the emission, and both the Purcell factor and the radiative rate enhancement exhibit an increasing tendency. In the case of the (i)-type coupled system optimized to enhance the excitation rate, a smaller distance from the nanorod center could result in a larger *QE*, but the achieved δ*R* would be smaller at both wavelengths (Figure 4a). Similarly, in case of the (ii)-type coupled system optimized to enhance excitation and emission simultaneously, the *QE* is decreased to the criterion by following a maximum close to the optimal dipole location, whereas the Purcell factor and δ*R* exhibit an increasing tendency (Figure 4b). In case of the (iii)-type system optimized to enhance the emission, the *QE* exhibits a maximum exactly at the optimal location at the emission wavelength, as a consequence, either a smaller or larger distance from the center would result in a smaller *QE* than the criterion. This indicates that it is not advantageous to tune the location, even though δ*R* would be larger at the excitation already in the case of de-locations smaller than 15 nm, whereas at the emission only for de-locations smaller than 7.5 nm (Figure 4c). Accordingly, the optimal dipole location corresponds to a compromised *QE* and δ*R* in all optimized coupled systems.

The sensitivity study on the dipole location predicts that the achievable radiative rate enhancement varies in the 30.40, 31.04 and 18.86 interval at the excitation, and in the 12.44, 27.39, 50.00 interval at the emission, when the Cy5 dye molecule with an optimal distance and orientation is moved from the Au NR center towards the tip in the (i)-, (ii)- and (iii)-type systems. The (ii)-type coupled system is the most sensitive to the Cy5 dye molecule location at the excitation wavelength, whereas it is intermediately sensitive at the emission to this parameter.

By varying the distance of the Cy5 dye molecule from the nanorod surface inside the 17.5 nm thick DNS strand, both the Purcell factor and the radiative rate enhancement monotonously decrease, and the quantum efficiency exhibits a maximum at a larger distance in the coupled systems optimized to enhance both phenomena, whereas it monotonously increases at both wavelengths in the systems optimized to maximize the radiative rate at 635 and 665 nm. The optimal distance is the threshold, above which the *QE* meets the criterion set at the emission (Figure 4d–f).

The sensitivity study on the dipole distance predicts that the achievable radiative rate enhancement varies in the 4.81, 4.76, 1.22 interval at the excitation, and in the 2.50, 5.5, 6.81 interval at the emission wavelength, when the Cy5 dye molecule with an optimal location and orientation is moved from the Au-NR surface towards the top of the DNS strand in the (i)-, (ii)- and (iii)-type systems. The (ii)-type coupled system is intermediately sensitive to the Cy5 dye molecule distance at both wavelengths. 

By varying the orientation of the Cy5 dye molecule with respect to the long axis of the nanorod, all of the Purcell factor, *QE* and radiative rate enhancement non-monotonously vary. At the optimal orientation, the *QE* meets the criterion set at the emission wavelength, and both the Purcell factor and the radiative rate enhancement exhibit an increasing tendency. However, a smaller tilting with respect to the long axis could result in a larger *QE*, but the achieved δ*R* would be smaller. Accordingly, the optimal orientation corresponds to a compromised *QE* and δ*R* (Figure 4g–i).

The sensitivity study on the dipole orientation predicts that the achievable radiative rate enhancement varies in the 18.61, 26.94, 19.91 interval at the excitation, and in the 6.55, 16.94, 34.45 interval at the emission, when the Cy5 dye molecule with an optimal location and distance is rotated in the (i)-, (ii)- and (iii)-type system. The (ii)-type coupled system exhibits the highest sensitivity to the Cy5 dye molecule orientation at the excitation, but only intermediate sensitivity at the emission wavelength.

### 3.2. Size Distribution and Spectral Properties of Gold Nanorods

Based on the TEM images, the mean value of the gold nanorods’ short and long axis is 14.6 nm ± 2.5 nm and 42.9 nm ± 7.1 nm, respectively. This corresponds to the mean aspect ratio of 2.94, which is close to the *AR* predicted by the optimizations. Two peaks are observable on the spectra of the nanorods, the local and global maximum at ~520 nm and 662 nm corresponds to the transversal and longitudinal resonance of the nanorod, respectively (Figure 5).

The transversal resonance is almost coincident with the particle plasmon resonance of gold according to the small short nanorod axes, whereas the longitudinal resonance indicates that the nanorods act as *λ*/2 mode antennas at 662 nm. The co-existence of resonance nearby the excitation and emission wavelengths of the Cy5 dye molecule is capable of resulting in an LDOS enhancement and in the improvement of both phenomena.

### 3.3. dSTORM Imaging of Au Nanorods Labelled with Cy5 Dye Molecule

The surface strands were bonded to the Au nanorods with high specificity via the thiol group. After being added to the sample, the probe strands predominantly bonded to the surface. This hybridization results in a stable double stranded structure. dSTORM images were captured using both oligonucleotide spacers listed in Table 1. Figure 6 and Figure 7 depict the comparative evaluation of the measured data.

It was assumed that during the STORM measurements, the Au nanorods laid parallel to the surface of the cover slip and were stable (Figure 6a). Despite the high specificity, a small amount of probe strands either attached to the surface of the cover slip or moved freely in the buffer, providing positive false localizations, and increased the background of the STORM image. The accepted localizations were arranged based on their fitted Gaussian peaks and only the most intense ones with peak values larger than 3500 counts were selected for further evaluations (orange bars in Figure 6b,e). This threshold was set as it is higher than the double value of the average peak intensity of the individual, unbonded dye molecules (≈1500 counts). Therefore, we can assume that the filtered bright spots were the images of fluorescence enhanced Cy5 dye molecules bounded to the gold nanorods, and the overlapping but unenhanced localizations were discarded. The histogram of the FWHMs of the fitted Gaussian PSF (orange bars in Figure 6c) proves that the selected bright spots correspond to the most perfectly focused Cy5 dye molecules (FWHM ≈ 1 pixel). The selected localizations in both cases (27 nt and 53 nt long spacers) have the highest, at a <6 nm Thompson precision as marked by the orange bars in Figure 6d,f [45]. The number of selected localizations using the longer (53 nt ≈ 17.5 nm) spacer dropped, showing that the dye molecules are too far from the Au NR, and fluorescence enhancement is reduced significantly.

Individual gold nanorods were identified with density based spatial clustering of applications with noise (DBSCAN) cluster analysis of the accepted localizations [46]. This algorithm requires two input parameters: a minimum number of points that forms a cluster (*N*) and the maximum distance between two adjacent points (*ε*). Clusters with *ε* = 160 nm and *N* = 8 set values were considered as footprints of plasmonically enhanced fluorescent Cy5 dye molecules on gold nanorods. Figure 7a,b or e,f depict the representative dSTORM images of isolated nanorods labelled with 27 nt and 53 nt long oligonucleotides, respectively. The nanorods are well isolated, no additional clusters can be found in the depicted 320 × 320 nm^2^ area.

Each of these nanorod features two hot spots, which are separated by 30–40 nm, and identify the two apexes of the nanorods. Figure 7c,d or g,h show the histogram of the mean and maximum fluorescence enhancement values using the 27 nt and 53 nt long spacers, respectively. The signal of the unbonded individual dye molecules was determined using the average value of localizations, which do not belong to any clusters. The normalization factors were 3639 and 3963 counts with the 27 nt and 53 nt long oligonucleotides, respectively. The significant difference between the numbers of identified nanorods (260 versus 11) proves that fluorescence enhancement is more significant, when the shorter (27 nt) spacer is applied. In this case, the maximum value of enhancement was found to be 20 and showed an exponential decay, whereas the mean value was typically below 10. When the longer (53 nt) linker is used, the ranges are identical (Figure 7g–h) but due to the reduced number of identified rods, the distribution cannot be resolved. This is in agreement with the distance dependence of fluorescence enhancement presented in Figure 4d–f.

We would like to emphasize that the Cy5 dye molecule fluorescence emission is enhanced by the plasmonic resonance of the gold nanorod and exhibits a well-defined directivity in contrast to the rotary photoluminescence of tiny gold nanoparticles, which originates from the anisotropic response of composing polycrystalline constituents [47]. However, due to the spectral overlap, the applied fluorescence microscope cannot distinguish photons emitted by the NRs or the fluorescence dye. The intensity of the autofluorescence of NRs was found to be significantly lower than the enhanced fluorescence signal of the dye molecules, therefore, its contribution to the fluorescence signal was negligible. Moreover, the autofluorescence signal of the NRs is static, and can be detected on all frames. The evaluation algorithm selected frames that showed only the Au NR signal (no blinking dye molecules) and used their average as a static fluorescence background. This static background was subtracted before the localization step. Despite this subtraction procedure, the localizations are probably shifted towards the center of the Au NRs caused by the spectral overlap, and the distance between the two hot spots are smaller than the real value. We did not use additional compensation/calibration procedures to fix this issue, and the measured fluorescence signal is the coherent sum of the enhanced fluorescence of the Cy5 dye molecule and the Au NR plasmon resonance. The two hot spots were only used to identify the NRs, whereas their exact separation was not a critical parameter. The real merit function was the intensity values of the blinking events.

## 4. Conclusions

In our present study, the synergy of the design and optimization of the coupled fluorescent molecule–plasmonic nanoparticle systems, the controlled preparation and functionalization of plasmonic nanoparticles and the state-of-the-art dSTORM monitoring made it possible to prove that the level of fluorescence enhancement predicted theoretically is achievable experimentally.

Namely, (i) the coupled Cy5 dye molecule and gold nanorod configurations optimal to enhance excitation and emission were designed, and moreover, it was shown that the highest simultaneous excitation and emission enhancement can be ensured by tuning the plasmonic resonance peak in between the Cy5 dye molecule excitation and emission wavelengths, (ii) Au NRs with a desired geometry were synthesized, (iii) Cy5 dye molecules were aligned at a desired distance from the Au NRs’ surface via hybridization of the Cy5-labelled oligonucleotide, and (iv) dSTORM was capable of detecting individual molecules that are stochastically on-state around individual Au NRs, thus single on-state-molecule detection proved that an emission enhancement is reached at the level predicted theoretically for individual dipolar emitters at analogue distances from individual Au NRs. The explanation of the good agreement between theory and experiment is that only those Cy5 dye molecules were observable by dSTORM, which approximated the optimal configurations.

We must also mention that the applied localization code is computationally intensive, and uses different thresholds, judging and selection steps. Therefore, localization techniques can introduce artifacts and the final super-resolution images can be misinterpreted. In this manuscript, the fluorescence intensity of individual dye molecules bonded to the NRs were normalized to unbonded ones. The measured intensity values cover a relatively wide range because fluorescence enhancement depends on the location, distance and orientation of the dye molecules relative to the Au NR. In the standard dSTORM measurements, we can only determine the intensity of the blinking events. An intensity threshold used to separate the brightest localizations can introduce data selection. However, we believe that due to the normalization procedure, the final quantitative evaluation clearly shows the difference between the two evaluated samples and confirms that fluorescence enhancement is significantly larger using the shorter 27 nt long spacer. The measured larger fluorescence enhancement is in agreement with the larger enhancement predicted theoretically.

## Figures and Tables

**Table 1:** TMTr-Thiol-ModifierC6 (SH-C6) and Cy5-fluorescent label were coupled by phosphoramidite chemistry to the 5′-end of oligonucleotides on the solid-phase.

Thiol-DNA	SH-C6-AATCTGTATCTATATTCATCATAGGAAACACCAAAGATGATATTTTCTTTAAT
Cy5-DNA 17.5 nm	Cy5-ATT AAAGAAAATATCATCTTTGGTGTTTCCTATGATGAATATAGATACAGATT
Cy5-DNA 8.9 nm	ATTAAAGAAAATATCATCTTTGGTGT + Cy5-TTCCTATGATGAATATAGATACAGATT

**Table 2:** Optical responses at excitation and emission wavelength and geometrical parameters of the optimized systems.

	Excitation (635 nm)		Medial (650 nm)		Emission (665 nm)	
	635 nm	665 nm	635 nm	665 nm	635 nm	665 nm
***QE* (%)**	13.4	25.8	9.7	24.75	8.4	27.5
***Purcell Factor***	44.4	15.4	47.8	24.2	14.7	19.8
**δ** ***R***	6.0	4.0	4.6	6.0	1.2	5.4
**δ** ***D*** **= *D*/*D*_0_**	1.4	0.7	0.7	0.7	0.5	0.6
**δ** ***D*** **× δ** ***R***	8.1	2.9	3.3	4.3	0.6	3.5
***P*** **_x_**	23.6		27.6		6.7	
***P*** **_x_** **× δ** ***D*** **_exc_** **× δ** ***D*** **_em_**	23.1		14.2		2.2	
***η*** **_outcoupling_**	0.98		0.51		0.32	
***a*_s_ (nm)**	20.1		20.2		21.9	
***a*_L_ (nm)**	37.7		39.8		44.6	
***AR***	1.87		1.97		2.04	
**Location *x* (nm)**	9.4		16.6		19.3	
**Distance *d* (nm)**	9.5		9.7		10.2	
**Orientation *φ* (°)**	53.0		21.7		10.3	
